# Microstructure and Mechanical Properties of TiN–TiB_2_–hBN Composites Fabricated by Reactive Hot Pressing Using TiN–B Mixture

**DOI:** 10.3390/ma14237198

**Published:** 2021-11-26

**Authors:** Qianglong He, Tian Tian, Shi Tian, Wenchao Guo, Yunwei Shi, Aiyang Wang, Hao Wang, Weimin Wang, Zhengyi Fu

**Affiliations:** The State Key Laboratory of Advanced Technology for Materials Synthesis and Processing, Wuhan University of Technology, Wuhan 430070, China; shsqlhe@whut.edu.cn (Q.H.); tiantianshs@whut.edu.cn (T.T.); shitian@whut.edu.cn (S.T.); whutguowc@whut.edu.cn (W.G.); 1917069803@whut.edu.cn (Y.S.); shswangh@whut.edu.cn (H.W.); zyfu@whut.edu.cn (Z.F.)

**Keywords:** TiN–TiB_2_–hBN, sintering temperature, composition ratio, microstructure, mechanical properties

## Abstract

In this study, TiN–TiB_2_–hBN composite ceramics were prepared via reactive hot pressing using TiN and amorphous B powders as raw materials. Different sintering temperatures and composition ratios were studied. The results show that the 70 vol% TiN–17.6 vol% TiB_2_–12.4 vol% hBN ceramic composites obtained ideal comprehensive properties at 1600 °C. The relative density, Vickers hardness, bending strength, and fracture toughness were 99%, 11 GPa, 521 MPa, and 4.22 MPa·m^1/2^, respectively. Densification was promoted by the highly active reaction product TiB_2_, and the structural defects formed in the grains. Meanwhile, the good interfacial bonding between TiN and TiB_2_ grains and the uniform dispersion of ultrafine hBN in the matrix contributed to the excellent bending strength. Moreover, the toughening mechanism of crack deflection and grain pull-out improved the fracture toughness.

## 1. Introduction

TiN gets a lot of attention owing to its excellent properties. As a high hardness and refractory nitride compound of group IVB transition metal, TiN has a hardness of 18 GPa and a melting point of 2950 °C [1,2]. Under high-temperature conditions, TiN still has high wear and excellent corrosion resistance, mainly attributed to the covalent bonding of materials [3,4]. In addition, TiN has a good thermal and electrical conductivity, similar to those of metals [5,6,7]. Titanium boride (TiB_2_) is very similar to titanium nitride, both of which are covalent refractory compounds and essential substrates of cermets. Some properties of TiB_2_, for instance Vickers hardness, thermal, and electrical properties, are better than those of TiN [8,9,10]. Thus, TiB_2_ has the potential to enhance TiN. In conclusion, TiN–TiB_2_ composite ceramics have a good application prospect in the fields of wear parts, aircraft engine components, cutters, and armors. Additionally, it has good prospects for molten salt electrolysis and high-performance power systems [11].

The strong covalent bonds of transition metal nitrides and borides contribute to a high melting point and low self-diffusion coefficient, making the densification of monolithic TiN and TiB_2_ very difficult [12,13,14]. In addition, the oxide impurities on the surface of the powder particles can promote grain coarsening, which further hinders densification [15]. Kitiwan et al. showed that the sintering temperature of TiN–TiB_2_ multiphase ceramics was lower than that of TiN and TiB_2_ single-phase ceramics [16,17]. In addition, studies have shown that, in hard refractory carbides, nitrides, and borides, the presence of hexagonal boron nitride or graphite with layered structures can significantly enhance the machining property and thermal stability [18,19,20,21]. The conventional process for preparing dense transition metal nitrides or borides involves introducing hexagonal boron nitride (hBN) powder into the matrix to form a composite powder by ball milling. Nonetheless, hBN is hard to disperse uniformly in ceramic matrix, which will damage the comprehensive properties of the materials [22]. Moreover, commercial TiB_2_ powders generally have relatively large particle sizes, and a large number of studies have shown that advanced sintering methods such as spark plasma sintering or reactive hot-pressing help prepare dense transition metal nitrides and borides.

Considering the above factors, in this study, TiN–TiB_2_–hBN composite ceramics were fabricated by TiN–B reaction sintering, and their microstructure and mechanical properties were explored.

## 2. Experimental Procedure

### 2.1. Raw Materials

Commercially available micron-sized TiN powder (d_50_ = 2~10 μm, purity ≥ 99.5%, China), and amorphous boron powder (d_50_ ≤ 2 μm, purity ≥ 95%, China), were used as the raw materials of reactive sintering to prepare TiN–TiB_2_–hBN composite ceramics. The synthesis reaction of TiN–TiB_2_–hBN is given as:

TiN + 3B → TiB_2_ + hBN (1)

The TiN–TiB_2_–hBN composite ceramics prepared by different compositions of TiN and amorphous boron are listed in Table 1.

**Figure 1:**
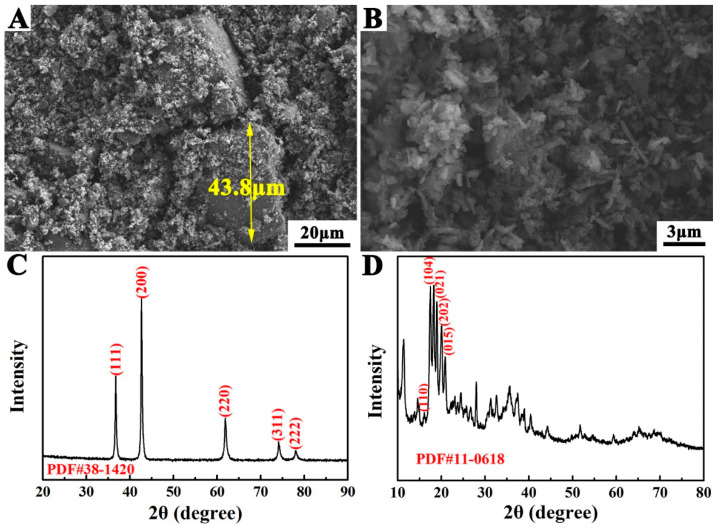
SEM images and XRD patterns of TiN (**A**,**C**) and amorphous Boron (**B**,**D**).

Figure 1A,C shows the SEM image and XRD pattern of TiN, and it was observed that the particle size of TiN varied widely. The smallest particle size of TiN was in the submicron scale, whereas the largest particle size of TiN was over 40 μm. No impurity peak could be observed from the corresponding XRD pattern; in addition, the relatively broad peaks indicated the presence of a certain proportion of submicron particles in TiN powders. Figure 1B,D shows that the amorphous boron powder had a small particle size and a rough surface. Moreover, several diffraction peaks of crystalline boron were observed in the corresponding pattern, indicating the presence of a certain proportion of crystalline boron in amorphous boron powder.

### 2.2. Sample Preparation

The TiN–B powder mixtures were weighed and placed in a polyethylene jar with ethanol (200 mL) and SiC balls (W_ball_:W_powder_ = 5:1); and then ball-milled for 2 h, using a high-speed planet mill (QXQM-4L, Tianchuang Powder Technology Co., Ltd, Changsha, China) at a rotary speed of 300 rpm. The slurries were dried at 65 °C for solid–liquid separation in a vacuum rotating evaporator (R, SENCO Technology Co., Ltd, Shanghai, China), and then transferred into a vacuum drying equipment (DZF-6050, Jinghong Laboratory Equipment Co., Ltd, Shanghai, China) to remove the residual ethanol. Afterwards, the as-received powder mixtures were sieved through a 200-mesh sieve to minimize the powder agglomeration, and the powder mixtures were poured into a cylindrical graphite die with an inner diameter of 48 mm; further, 0.2 mm-thick graphite foil lining was used to prevent the powders from contacting directly with the graphite die. Finally, the bulk sample was sintered using a hot-pressing furnace (916G-G, Thermal Technology LLC, Santa Rosa, CA, USA) at sintering temperature for 0.5 h with a pressure of 30 MPa. The furnace was heated to 1400 °C at 20 °C/min in vacuum, and then, after dwelling for 0.25 h to remove the volatile impurities, we continued to raise the temperature at 10 °C/min to the sintering temperature in an argon atmosphere. The pressure remained at 10 MPa until the temperature reached 1400 °C, and with the temperature rising to sintering temperature, the pressure rose to 30 MPa at a constant rate.

### 2.3. Characterization

The final density of the bulk sample was measured using the Archimedes method. The specimens used for evaluating the mechanical properties were machined by electrical discharge machining (EDM). The bending strength was measured by the three-point bending method (sample size: 3 × 4 × 36 mm^3^, span: 30 mm, loading rate: 0.5 mm/min). The fracture toughness (K_IC_) was measured via single-edge notched beam (SENB) method (sample size: 2.5 × 5 × 25 mm^3^; notch depth = 2~2.5 mm, span: 20 mm, loading rate: 0.05 mm/min). The bending strength and fracture toughness of the composites were measured by a ceramic test system (CMT6503, Meitesi Testing Technology Co., Ltd, Ji’nan, China), and the values were determined based on the measurements of 5 bars. The hardness was tested using a Vickers hardness tester (430SVD, Wilson, Boston, MA, USA) with a load of 1 kg for a dwelling time of 15 s on a polished specimen surface; the final value was the average of 7 indentations. The particle size distributions of TiN–B composite powder before and after ball milling were measured using a laser particle analyzer (Mastersizer2000, Malvern Instruments Co., Ltd, Malvern, UK). The phase components and microstructure were investigated by X-ray diffraction (XRD; Empyrean, PANalytical B.V., Eindhoven, The Netherlands), scanning electron microscopy (SEM; 3400, Hitachi, Tokyo, Japan), and transmission electron microscope (TEM; F200s, Thermo Fisher Scientific, Waltham, MA, USA), respectively.

## 3. Results and Discussion

### 3.1. Influence of Ball Milling on TiN–B Powder Mixture

**Figure 2:**
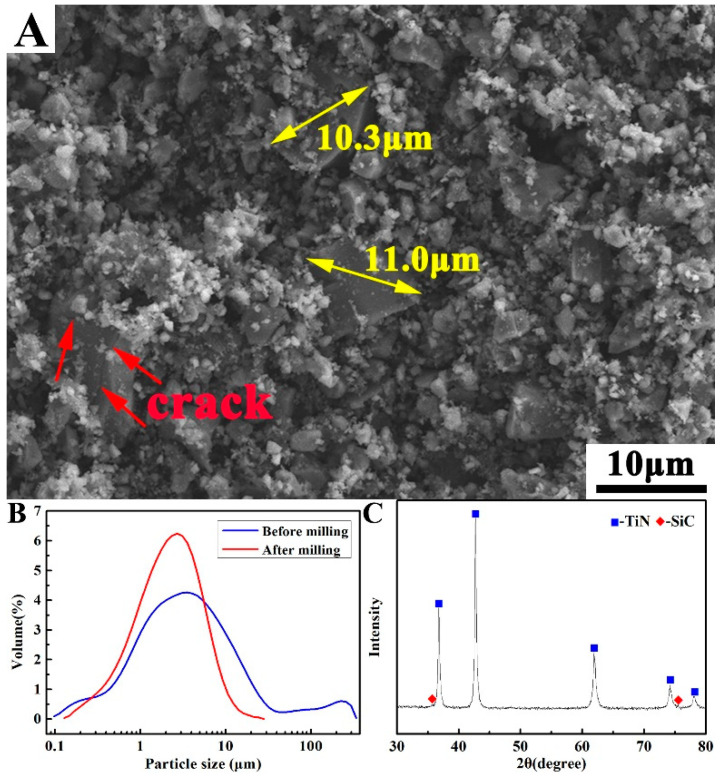
(**A**) SEM image, (**B**) particle size distribution, and (**C**) XRD pattern of 94.7 wt. % TiN–5.3 wt. % B powder mixture after ball milling.

As observed in Figure 2A, after ball milling for 2 h at 300 r/min, the large particles (over 40 μm) in the TiN powders have completely disappeared. There are noticeable cracks on the large TiN particles (marked by red arrow), which indicates that the particles were crushed during ball milling. The relatively large particle size in the TiN–B powder mixture after ball milling was approximately 10 μm. From the particle size distribution after ball milling, as shown in Figure 2B, it can be seen that the supersized aggregates (>100 μm) in the powder mixture were effectively dispersed, and the large TiN particles were effectively refined. Silicon carbide (SiC) balls were used in the ball milling process, and the hardness of SiC was greater than that of TiN; therefore, TiN particles, the main component of the powder mixture, were effectively refined during ball milling. Consequently, a small amount of SiC was introduced into the powder mixture, as shown in the XRD pattern of the powder mixture (Figure 2C).

### 3.2. Influence of Sintering Temperature on the Microstructure and Mechanical Properties of TiN–TiB_2_–hBN Composite Ceramics

**Figure 3:**
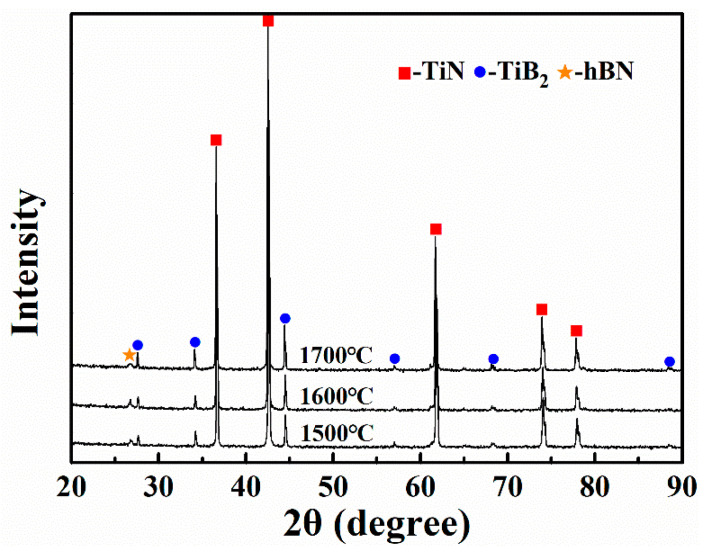
XRD patterns of the TB2 samples sintered at different temperatures.

Figure 3 shows the XRD patterns of the samples obtained by sintering TiN–B powder mixtures at 1500, 1600 and 1700 °C. TiN–TiB_2_–hBN composite ceramics were obtained at three different sintering temperatures, and no impurity phase was produced. Figure 3 shows that, when the sintering temperature was 1700 °C, the diffraction peak of TiB_2_ of the sample was enhanced to some extent, while the intensity of the diffraction peak of hBN was weakened. The occurrence of Equation (2) caused the change in component content in the sample and further caused the change in XRD peak intensity. Therefore, the test results of XRD proved the rationality of the above prediction. Kitiwan et al. reported a slight reaction between TiN and hBN at 1800 °C. However, in this study, the oxide layer on the TiN surface was removed after reaction with B, and hBN was synthesized by in situ reaction. Therefore, TiN and hBN in this study have higher reactivity than that of commercial TiN and hBN; consequently Equation (2) occurs only at 1700 °C.

TiN + 2BN → TiB_2_ + 3/2N_2_ (g) (2)

**Figure 4:**
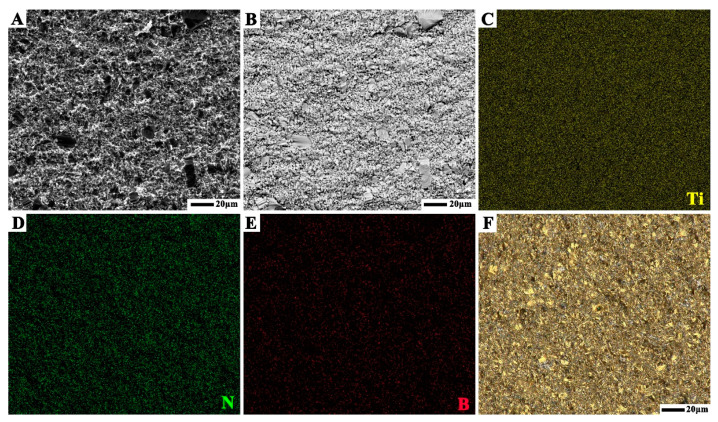
(**A**) SEM and (**B**) BSE images of TB2 sintered at 1600 °C; distribution of (**C**) Ti, (**D**) N, and (**E**) B elements; (**F**) metallographic image of TB2 sintered at 1600 °C.

In order to see the arrangement of individual phases, SEM images in BSE contrast was carried out, as shown in Figure 4A,B. The distribution of large size TiN grains can be seen, but since the atomic masses of TiN and TiB_2_ are very similar, it is difficult to further identify the distribution of individual phases. It can be seen from Figure 4C–E that the absent area of Ti is the hBN phase, the absent area of N is the TiB_2_ phase, and the absent area of B is the TiN phase. In addition, as shown in Figure 4F, different phases can be identified through optical images. The yellow region is TiN, gray region is TiB_2_, and the white region is hBN. It can be concluded that the grain size of TiN is large, while that of TiB_2_ and hBN is fine, and TiN, TiB_2_, and hBN are uniformly distributed in the ceramic composites.

**Figure 5:**
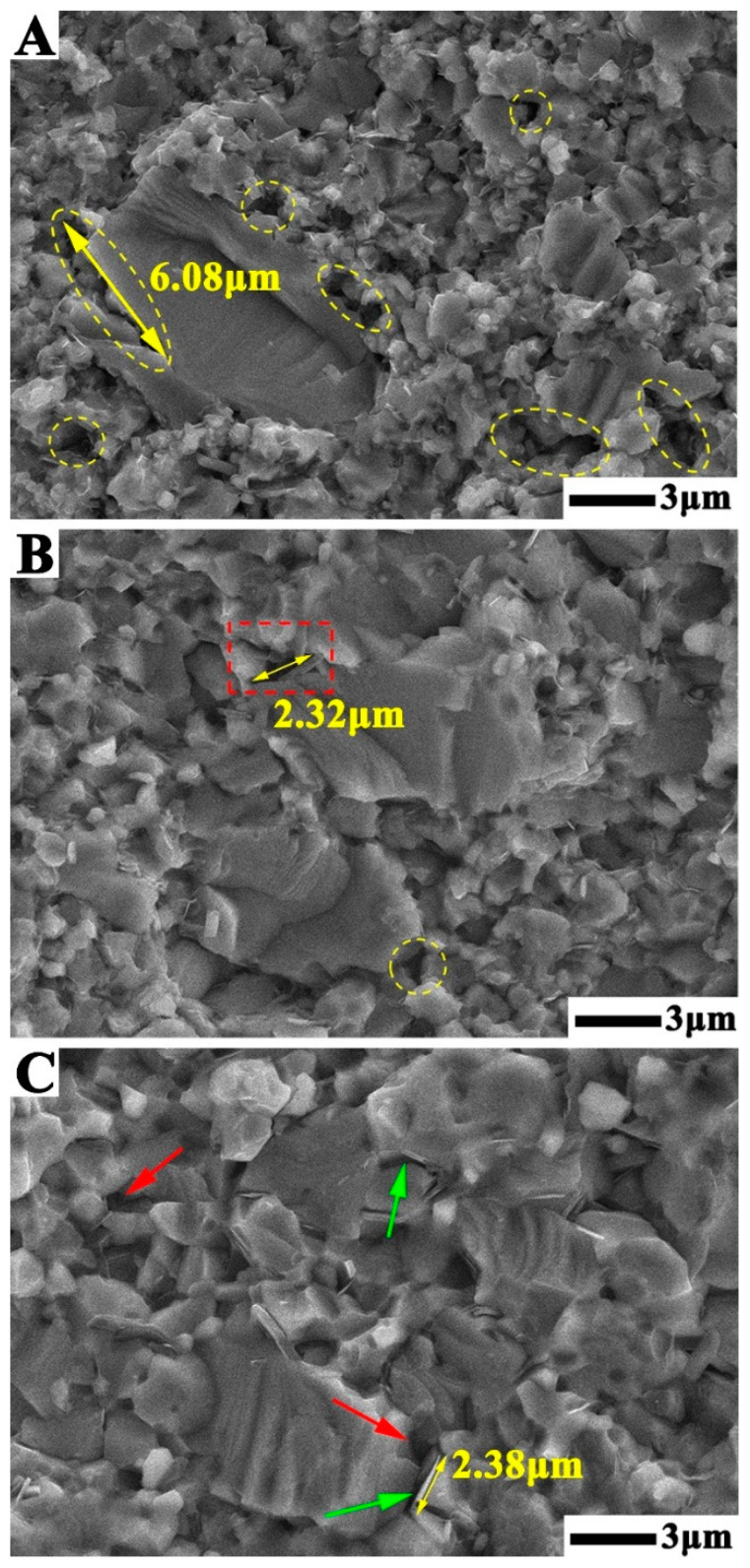
SEM images of the fractured surface of TB2 sintered at (**A**) 1500 °C, (**B**) 1600 °C, and (**C**) 1700 °C.

Holes with irregular shapes were formed, as shown by yellow dotted circles in Figure 5A, indicating that these areas have not undergone complete densification owing to the low sintering temperature. The yellow dotted circle in Figure 5B indicates the holes similar to those in Figure 5A, while the red dotted box indicates a frame structure in which the hole was difficult to eliminate due to hindrance by the plate-like hBN. It was evident that when the sintering temperature increased to 1600 °C, the density of the TiN–TiB_2_–hBN composite ceramic was significantly improved. When the temperature increased to 1700 °C, holes with regular shapes were formed (marked by the red arrows in Figure 5C), which can be attributed to TiB_2_ or TiN grains being pulled out. At this stage, due to incomplete densification the holes could not be observed entirely; only the gaps formed by plate-like hBN and surrounding grains (marked by the green arrow) were visible, which were created by the excessive thermal expansion of hBN grains along the c-axis [23].

**Figure 6:**
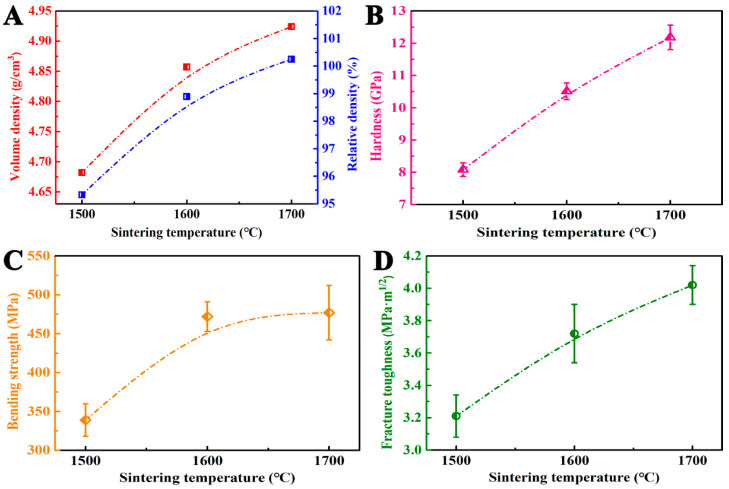
(**A**) Volume and relative densities, (**B**) Vickers hardness, (**C**) bending strength, and (**D**) fracture toughness of TB2 sintered at different temperatures.

As shown in Figure 6A, the bulk and relative densities of sample TB2 increased with the increase in sintering temperature. When the sintering temperature was 1500 °C, several holes could not be eliminated because of the insufficient sintering driving force, and the relative density of the sample was only 95.3%. The measured relative density data were in good agreement with the SEM observation. When the sintering temperature was increased to 1600 °C, the densification degree of the sample was greatly improved, and the relative density reached 98.9%, which was close to the level of complete densification; however, a few pores remained and resulted in the fractured surface. When the sintering temperature continued to rise to 1700 °C, the relative density of the sample was measured as high as 100.3%. Figure 5C shows that there are still some gaps in the sample due to the mismatch of the thermal expansion coefficient between hBN and the surrounding grains. Therefore, the sample was not completely dense, and the true relative density was less than 100%. Hence, it can be inferred that the above phenomenon is affected by reactions that change the composition of the sample, resulting in a deviation in the values of the relative density. Studies have shown that when the sintering temperature reach a certain degree during the synthesis of TiN–hBN composites, TiN slightly reacts with hBN, forming a small amount of TiB_2_ in the composites [24]. The reaction process is shown in Equation (2).

TiN (5.24 g/cm^3^), TiB_2_ (4.52 g/cm^3^), and hBN (2.29 g/cm^3^) were used to calculate the theoretical densities. According to Equation (2), for the case of a complete dense-packed reactants powder mixture, the theoretical density was 3.33 g/cm^3^. Meanwhile, the theoretical density of the products is the density of TiB_2_ (4.52 g/cm^3^). Therefore, the difference in theoretical densities used in calculating the relative density was too small, resulting in a relative density of 100.3%.

Figure 6B shows that the Vickers hardness of sample TB2 improved with the increase in sintering temperature. When the sintering temperature was 1500 °C, the Vickers hardness value of the sample was 8.1 GPa because the densification degree of the sample was only 95.3%. When the sintering temperature was increased to 1600 °C, the relative density of the sample was significantly increased; consequently, the Vickers hardness of the sample also increased significantly to 10.5 GPa. When the sintering temperature was further increased to 1700 °C, the densification degree of the sample increased slightly; however, the occurrence of Equation (2) between TiN, a relatively low Vickers hardness material (18 GPa), and the soft phase hBN resulted in the formation of TiB_2_ with a high Vickers hardness (29 GPa). Therefore, the relative density of TB2 was significantly improved.

Pores are the source of crack initiation and can promote crack propagation. According to Griffith’s theory, the size of a defect largely determines the bending strength of ceramic composites. As shown in Figure 5, the maximum defect size in the sample sintered at 1500 °C was 6.08 μm, which was formed by the pores present in the low-density sample. With the increase in sintering temperature, the pores in the sample were significantly reduced, and the maximum defect size was reduced to 2.32 μm. When the sintering temperature was increased to 1700 °C, the pores disappeared. However, the hBN grains grew substantially, and the gap formed between the hBN grains and surrounding grains reached 2.38 μm. Therefore, the samples sintered at 1500, 1600 and 1700 °C had the bending strength of 339 MPa, 472 MPa, and 477 MPa, respectively. It can be seen that the changing rule of the bending strength of the samples (Figure 6C) is highly consistent with the changing rule of the defect size in the samples. As shown in Figure 6D, the fracture toughness of the sample improved with the increase in sintering temperature because the density of the sample increased with increasing sintering temperature. In addition, TiB_2_ and hBN grains grow as the sintering temperature increases; this induces more crack deflection and consumes more crack propagation energy, and as a result, the fracture toughness of the samples increases.

Based on the above results, it can be concluded that 1600 °C is an appropriate sintering temperature for the in situ reaction of TiN–B systems to synthesize TiN–TiB_2_–hBN composite ceramics with compact microstructures, excellent properties and no additional reactions.

### 3.3. Influence of Composition Ratio on the Microstructure and Mechanical Properties of TiN–TiB_2_–hBN Composite Ceramics

**Figure 7:**
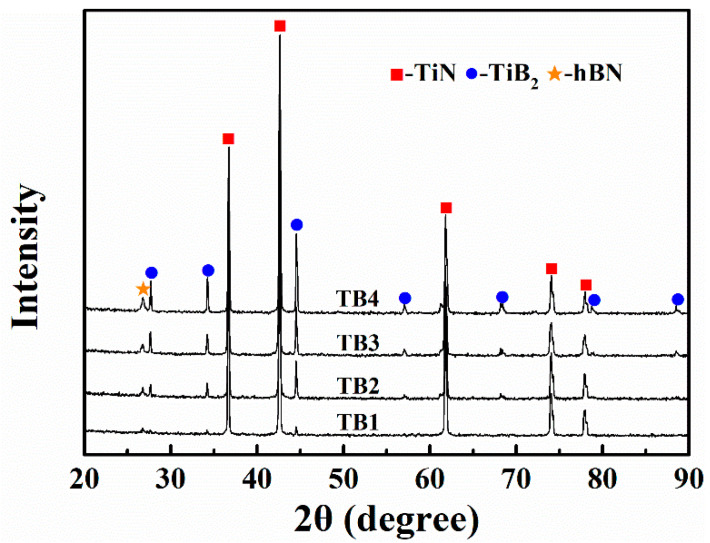
XRD patterns of bulk samples TB1, TB2, TB3, and TB4 sintered at 1600 °C.

Figure 7 shows the XRD patterns of the bulk samples with different TiB_2_–hBN contents. TB1, TB2, TB3, and TB4 are all composed of TiN, TiB_2_, and hBN, without other impurity phases. Figure 7 shows that the increase in TiB_2_ and hBN contents led to a continuous increase in the intensity of the two corresponding diffraction peaks.

**Figure 8:**
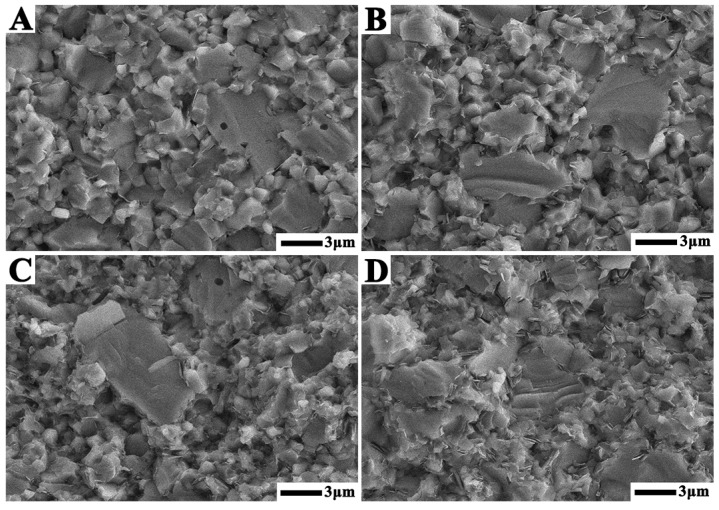
SEM images of the fracture surface of (**A**) TB1, (**B**) TB2, (**C**) TB3, and (**D**) TB4.

Figure 8 shows the section morphologies of TiN–TiB_2_–hBN composite ceramic samples with different TiB_2_–hBN contents prepared at 1600 °C. The figure shows that TB1, TB2, TB3, and TB4 have dense microstructures. Owing to the large particle size of TiN raw material, the TiN in TiN–TiB_2_–hBN samples retained a large grain size. Meanwhile, TiB_2_ and hBN were obtained by in situ reaction; therefore, TiB_2_ and hBN had small grain sizes and were uniformly dispersed in TiN matrix.

**Figure 9:**
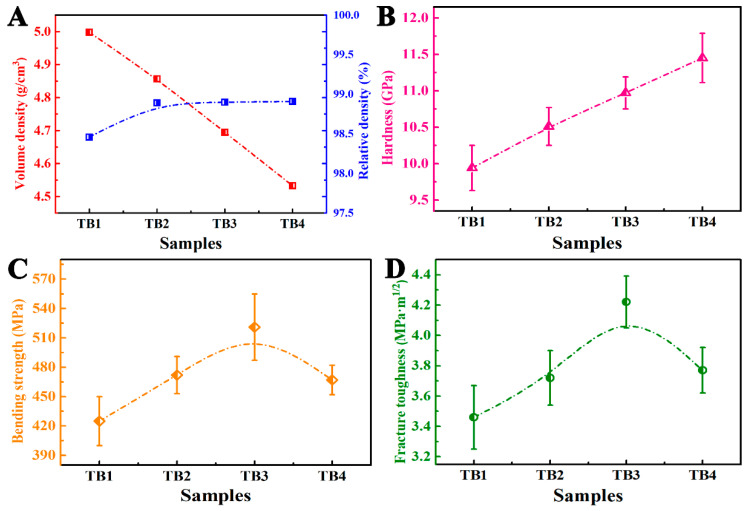
(**A**) Volume and relative densities, (**B**) Vickers hardness, (**C**) bending strength, and (**D**) fracture toughness of TB1, TB2, TB3, and TB4.

As shown in Figure 9A, the volume density of TiN–TiB_2_–hBN composite ceramics decreased with the increase in TiB_2_–hBN contents because the volume densities of TiB_2_ (4.52 g/cm^3^) and hBN (2.29 g/cm^3^) are lower than that of TiN (5.24 g/cm^3^). In addition, the relative density test results show that almost all four samples have reached the degree of complete density. When the TiB_2_–hBN content was 10 vol%, the relative density of the sample was approximately 98.5%, and when the content of TiB_2_–hBN was greater than or equal to 20 vol%, the relative density of the sample was approximately 99%. TiB_2_ and hBN are synthesized by in situ reactions and have high sintering activity. Meanwhile, the layered and uniformly dispersed hBN can act as a lubricant and diffusion channel in the sintering process [25,26]. Therefore, when the TiB_2_–hBN content is low, the relative density of TiN–TiB_2_–hBN composite cannot be high. Figure 9B shows that the Vickers hardness of the TiN–TiB_2_–hBN composite ceramics increased with the decrease in TiN content. The hBN produced by in situ reaction has a small grain size and good dispersion, which has a positive effect on the hardness of the ceramic matrix. The hardness of TiB_2_ synthesized by the reaction was as high as 29 GPa. According to the mixture rules, an increase in the TiB_2_ content improves the hardness of the ceramic composites. The grain size of TiB_2_ is fine. Fine grains make grain boundaries more concentrated, which means that moving dislocations are more likely to encounter grain boundaries. The accumulation of dislocation at grain boundaries makes deformation more difficult [27]. Therefore, the Vickers hardness of TiN–TiB_2_–hBN composite ceramic increased with an increase in the TiB_2_–hBN content. Figure 9C shows the bending strength of TiN–TiB_2_–hBN composite ceramics. When the TiB_2_–hBN content was less than 30 vol%, the bending strength of the TB composites increased with an increase in the TiB_2_–hBN content; this is because the increase in TiB_2_–hBN content suggests an increase in TiN consumption, and the decrease in TiN grain size decreases the initial microcrack size in the samples, thus increasing the bending strength of the TB composites. When the content of TiB_2_–hBN reached 40 vol%, the hBN content was as high as 16.5 vol%, and some hBN particles agglutinated, resulting in an increase in the initial microcrack size in the sample, thus reducing the bending strength of the TiN–TiB_2_–hBN composite ceramics.

Goto et al. prepared 26 vol% TiN–61 vol% TiB_2_–13 vol% hBN composite ceramics by commercial TiN, TiB_2_, and hBN powders at 1700 °C and 100 MPa. The relative density, Vickers hardness, and fracture toughness of the sample reached 96%, 20.1 GPa, and 4.3 MPa·m^1/2^, respectively [17]. However, the bending strength was not reported. The main phase in the literature is TiB_2_, while the main phase in the present work is TiN. The hardness of TiB_2_ is much higher than that of TiN, so the hardness value reported in the literature is higher. TiN–TiB_2_–hBN composite powder obtained by reaction has better sinterability. Therefore, a higher densification degree (99%) can be obtained under milder sintering conditions in present work.

### 3.4. Strengthening and Toughening Mechanism of TiN–TiB_2_–hBN Composite Ceramics

**Figure 10:**
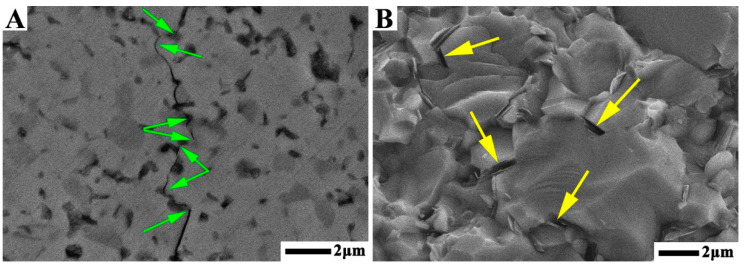
(**A**) Crack propagation path of polished surface and (**B**) fracture surface of TB2.

The propagation path of cracks and the fracture surface morphology were studied to investigate the toughening mode of TB composites. Figure 10A shows a backscattered electron (BSE) image of the crack propagation path on the polished surface of TB2. Different phases of the sample are identified depending on their atomic masses, where the light grey area in the BSE image is the TiN matrix, the dark grey area is TiB_2_, and the black area is hBN. The crack propagation shows that the primary toughening mechanism of TiN–TiB_2_–hBN composite ceramics was due to crack deflection, and most of the crack deflection occurred at the interface between different phases due to weak interface bonding caused by the thermal expansion coefficients mismatch of different phases. In addition, the yellow arrow in Figure 10B shows another toughening method for TB composites, that is, the pull-out of hBN grains.

In Figure 9D, when the TiB_2_–hBN content was less than 30 vol%, the fracture toughness of the TB composites increased with an increase in the TiB_2_–hBN content. This variation in toughness is because with an increase in the TiB_2_–hBN content, the probability of deflection during crack propagation in the samples increased. When the TiB_2_–hBN content was increased to 40 vol%, the crack deflection frequency was saturated, and the bond strength between different phases was further weakened, which led to a decrease in energy consumption during crack propagation and a decrease in the fracture toughness of the TB composites.

**Figure 11:**
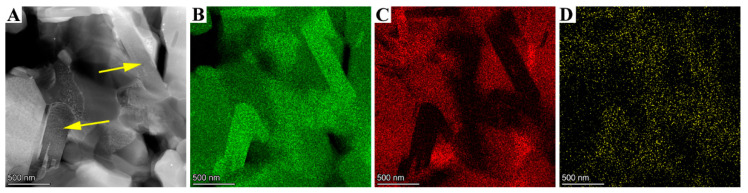
TEM image of (**A**) TB2 and element distribution map of (**B**) Ti, (**C**) N, and (**D**) B.

Figure 11A shows that long rod-like grains are visible in the TiN–TiB_2_–hBN composite ceramic. The elemental distribution results show that the long rod-like grains are the aggregation region of Ti and B, and the depleted region corresponds to N; therefore, it can be concluded that these long rod-like grains are TiB_2_. In general, these rod-like TiB_2_ grains have a toughening effect, which is crack deflection and bridging. Therefore, these rod-like TiB_2_ tend to improve the properties of TB composites [28].

**Figure 12:**
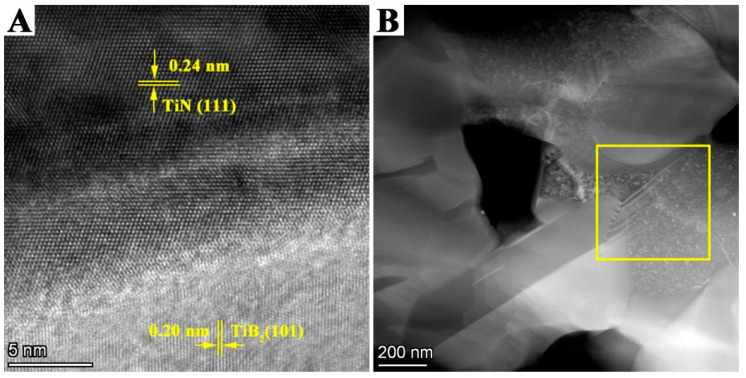
(**A**) HRTEM and (**B**) TEM images of TB2 composite.

Figure 12A shows that the interplanar distances of the two adjacent grains are 0.24 nm and 0.20 nm, which correspond to the (111) lattice plane of TiN and (101) plane of TiB_2_. It is evident that the interface of TiN and TiB_2_ is well integrated, which is the basis for the TiN–TiB_2_–hBN composite ceramics to attain good mechanical properties [29]. Figure 12B shows that the TB composite has structural defects of twins and stacking faults. Such structural defects not only increase the diffusion coefficient, improve mass transfer, and promote densification but also hinder crack propagation and improve fracture toughness as a sub-interface [30,31,32].

## 4. Conclusions

In this study, TiN–TiB_2_–hBN composite ceramics were fabricated through RHP sintering by TiN and amorphous boron powders. The effects of the sintering temperature and components on the microstructure and comprehensive properties of the samples were investigated. First, taking TB2 as the base sample, the effect of sintering temperatures was studied, and the results showed that 1600 °C is an appropriate sintering temperature for the in situ reaction sintering system of TiN–B. Then, TiN–TiB_2_–hBN composite ceramics with different TiB_2_–hBN contents were prepared at a sintering temperature of 1600 °C. The results showed that TB3 obtained the best comprehensive properties. The relative density, Vickers hardness, bending strength, and fracture toughness were 99%, 11 GPa, 521 MPa, and 4.22 MPa·m^1/2^, respectively. Meanwhile, the mechanisms promoting sintering, strengthening, and toughening were discussed.

## Figures and Tables

**Table 1:** Content of raw material and the composition of bulk samples.

Serial Number	Raw Materials (g)		Bulk Samples ( vol%)		
	TiN	B	TiN	TiB_2_	hBN
TB1	29.71	0.78	90	5.87	4.13
TB2	27.98	1.57	80	11.73	8.27
TB3	26.26	2.35	70	17.59	12.41
TB4	24.53	3.13	60	23.46	16.54

## Data Availability

The data presented in this study are available on request from the corresponding author.
